# Restrictive spirometric pattern and true pulmonary restriction in a general population sample aged 50 - 64 years

**DOI:** 10.1186/s12890-020-1096-z

**Published:** 2020-02-27

**Authors:** Kjell Torén, Linus Schiöler, Jonas Brisman, Andrei Malinovschi, Anna-Carin Olin, Göran Bergström, Björn Bake

**Affiliations:** 1 Occupational and Environmental Medicine, School of Public Health and Community Medicine, Sahlgrenska Academy, University of Gothenburg, Box 414, S-405 30 Gothenburg, Sweden; 2 Department of Occupational and Environmental Medicine, Sahlgrenska University Hospital, Gothenburg, Sweden; 3 Department of Medical Sciences, Uppsala University, Uppsala, Sweden; 4 Department of Molecular and Clinical Medicine, Institute of Medicine, Sahlgrenska Academy, University of Gothenburg, Gothenburg, Sweden; 5 Department of Medicine/Lung Medicine, Sahlgrenska Academy, University of Gothenburg, Gothenburg, Sweden

**Keywords:** Validity, Restrictive lung disease, RSP, TLC, Reference values, SCAPIS

## Abstract

**Background:**

There is low diagnostic accuracy of the proxy restrictive spirometric pattern (RSP) to identify true pulmonary restriction. This knowledge is based on patients referred for spirometry and total lung volume determination by plethysmograpy, single breath nitrogen washout technique or gas dilution and selected controls. There is, however, a lack of data from general populations analyzing whether RSP is a valid proxy for true pulmonary restriction. We have validated RSP in relation to true pulmonary restriction in a general population where we have access to measurements of total lung capacity (TLC) and spirometry.

**Methods:**

The data was from the Swedish CArdioPulmonary bioImage Study (SCAPIS Pilot), a general population-based study, comprising 983 adults aged 50–64. All subjects answered a respiratory questionnaire. Forced expiratory volume in 1 s (FEV_1_) and forced vital capacity (FVC) were obtained before and after bronchodilation. TLC and residual volume (RV) was recorded using a body plethysmograph. All lung function values are generally expressed as percent predicted (% predicted) or in relation to lower limits of normal (LLN). True pulmonary restriction was defined as TLC < LLN_5_ defined as a Z score < − 1.645, i e the fifth percentile. RSP was defined as FEV_1_/FVC ≥ LLN and FVC < LLN after bronchodilation. Specificity, sensitivity, positive and negative likelihood ratios were calculated, and 95% confidence intervals (CIs) were calculated.

**Results:**

The prevalence of true pulmonary restriction was 5.4%, and the prevalence of RSP was 3.4%. The sensitivity of RSP to identify true pulmonary restriction was 0.34 (0.20–0.46), the corresponding specificity was 0.98 (0.97–0.99), and the positive likelihood ratio was 21.1 (11.3–39.4) and the negative likelihood ratio was 0.67 (0.55–0.81).

**Conclusions:**

RSP has low accuracy for identifying true pulmonary restriction. The results support previous observations that RSP is useful for ruling out true pulmonary restriction.

## Introduction

True pulmonary restriction is synonymous with reduced total lung capacity (TLC) and is associated with a number of pathological conditions that either take up space in the thoracic cavity or restricts movements of the thoracic cage or diaphragm – for example interstitial lung diseases, pleurisy, lung edema, kyphosis, neuromuscular weakness and severe obesity. Measurements of TLC require relatively sophisticated equipment such as a body plethysmograph, or helium or nitrogen gas analyzers [1, 2]. These measurements are usually done in specialized lung function laboratories. In recent years, TLC has also been measured using inspiratory and expiratory chest computed tomography [3].

Conversely, dynamic spirometry can be done at low cost. The procedure is simple and widely used, but does not measure TLC. Consequently, there is a need for a spirometric algorithm that identifies reduced TLC with high diagnostic accuracy. It has been suggested that low vital capacity in the absence of airflow limitation could be used as a proxy for true pulmonary restriction. The proxy restrictive spirometry pattern (RSP) has been defined as forced expiratory volume in 1 s (FEV_1_)/forced vital capacity (FVC) ≥ 0.7 and FVC < 80% predicted [4]. Alternative definitions, based on the lower limit of normal (LLN), are becoming more widely used, and the most common of these is to define RSP as FEV_1_/FVC ≥ LLN and FVC < LLN [2]. Slow vital capacity (SVC) is usually larger than the FVC [5]. Hence, it would also be of interest to define RSP using SVC instead of FVC.

We have identified five studies investigating RSP in relation to static lung volumes [1, 6–9]. All five studies were based on patients referred for spirometry and lung volume determination by plethysmograpy or gas dilution and selected controls. Generally, reduced TLC was defined as <LLN. The sensitivity of RSP ranged from 68 to 100% and the specificity from 61 to 93% depending chiefly on the chosen cut-off values for vital capacity.

Some general population studies report using RSP as a proxy for true pulmonary restriction [10–14]. However, the prevalence of true pulmonary restriction in these general population studies is unknown, as TLC was not measured. Further, the applied definitions of RSP varied with regard to whether results reflect measurements before or after bronchodilation and how the cut-off values were defined, for instance based on the LLN or on percentage of predicted normal values. There is a lack of data from general populations regarding whether RSP is a valid proxy for true pulmonary restriction. In other words, studies are lacking where persons in a random general population sample have been investigated with both dynamic spirometry and static lung volumes, TLC.

The RSP phenotype has been linked to diabetes, metabolic syndrome and increased mortality and RSP is probably capturing a different phenotype but overlapping group in relation to true pulmonary restriction, low TLC [2].

Hence, there is a need to perform a general population study examining the validity of the proxy RSP in identifying true pulmonary restriction. We have performed a validation study in a general population sample, for which we had access to measurements of TLC using body box, residual volume (RV), and dynamic spirometry before and after bronchodilation. Hence, we will have the possibility to validate RSP for indicating true pulmonary restriction.

## Material and methods

Our data was from the pilot part of the Swedish CArdioPulmonary bioImage Study (SCAPIS Pilot), a Swedish general population-based study. For this initial pilot study, a randomly selected population sample of 2243 adults aged 50–64 years were invited to take part and 1111 agreed to participate [5, 15]. All persons answered an extensive respiratory questionnaire, including detailed items about smoking habits.

Dynamic spirometry, including FEV_1_, FVC and slow vital capacity (SVC) was performed before and 15 min after inhalation of 400 μg of salbutamol using a nose clamp with the subject in a sitting position. All accepted exhalations had a duration of > 6 s and a plateau on the curve the last second of the exhalation. Static lung volumes, TLC and RV, were determined by body plethysmography based on two measurements recorded after bronchodilation. There were daily calibrations of pressure, volume and flow. All procedures were performed according to American Thoracic Society (ATS)/European Respiratory Society (ERS) standards [16]. There were daily volume controls of the spirometer. All beta-agonist were withheld the day of the investigation. A Jaeger Master Screen pulmonary function (PFT) system (Vyaire, Mettawa, Illinois, US) was used for all measurements. Predicted values of FEV_1_, FVC and SVC were based on recently published local reference equations [17, 18]. Predicted values for TLC and RV were based on published equations [19]. For analysis of TLC, we additionally developed a local reference equation for TLC based on the never-smokers in the present study population without any respiratory symptoms (wheeze, dyspnea, chronic bronchitis) and without self-reported heart diseases. All lung function values are generally expressed as percent predicted (% predicted) or in relation to the LLN, using published locally equations [17, 18].

### Definitions

True pulmonary restriction was defined as TLC < LLN described as a *z* score < − 1.645 (i. e. a *z* score below the fifth percentile), using both the published equations of Quanjer et al., TLC < LLN_QUANJER_, and the local equation TLC < LLN_GOTHENBURG_, see “Statistics” [19].

Restrictive spirometric pattern (RSP) was in five different ways;

- RSP_LLN_ = FEV_1_/FVC ≥ LLN and FVC < LLN after bronchodilation
- RSP_0.7_ = FEV_1_/FVC ≥ 0.7 and FVC < 80% predicted after bronchodilation [17, 18].
- RSP_PREDIL_ = FEV_1_/FVC ≥ LLN and FVC < LLN before bronchodilation [17, 18].
- RSP_LLNSVC_ = FEV_1_/SVC ≥ LLN and SVC < LLN after bronchodilation [17, 18].
- RSP_0.7SVC_ = FEV_1_/SVC ≥ 0.7 and SVC < 80% predicted after bronchodilation [17, 18].

Asthma was defined as an affirmative answer to an item about physician-diagnosed asthma [20]. Dyspnea was defined as a modified Medical Research Council (mMRC) breathlessness score ≥ 2 [21].

Smoking was categorized into current smokers, former smokers, and never-smokers. Former smokers were defined as those who had smoked for at least 1 year but who had not smoked during the past 12 months. In this analysis current smokers and former smokers were categorized as ever-smokers.

### Statistics

All calculations were performed using SAS version 9.4 (SAS Institute, Cary, NC, USA). The local reference equation for TLC was computed by a linear regression model with height as a covariate, stratified by gender. The resulting equation for women was TLC = height (cm)*0.085–8.71, with residual standard deviation (RSD) = 0.56. For men the equation was TLC = height (cm)*0.102–10.93), with RSD = 0.83.

Specificity, sensitivity, positive and negative predictive values (PPV, NPV), positive and negative likelihood ratios (LR+ and LR-) were calculated, and 95% confidence intervals (CIs) were calculated using exact methods. Post-test probabilities of disease after positive and negative tests were assessed [22]. We calculated the percentiles of FVC, and sensitivity and specificity of RSP_LLN_ using increasing percentiles of FVC were plotted. Factors associated with disconcordance between true pulmonary restriction and RSP_LLN_ and RSP_LLNSVC_ were analyzed as odds ratios (OR), using multiple logistic regression. The variables age, body mass index, gender, smoking habits and RV were a priori selected as potentially associated with disconcordance.

## Results

Of the 1111 subjects, 128 were excluded because of incomplete data on smoking and dynamic spirometry or missing TLC measurements, resulting in a final study population of 983 subjects. Descriptive data on age, gender, smoking, lung function, and prevalence of asthma and dyspnea is shown in Table 1. The prevalence of true pulmonary restriction, TLC < LLN_QUANJER_, was 4.7% (*n* = 46), and when using our local equation, the prevalence of TLC < LLN_GOTHENBURG_ was 5.4% (*n* = 53). The prevalence of RSP_LLN_, RSP_0.7_, and RSP_PREDIL_ was, 3.4% (*n* = 33), 3.2% (*n* = 31), and 3.4% (*n* = 23), respectively. When applying SVC, the prevalence of RSP_LLNSVC_ and RSP_0.7SVC_ was 2.1% (*n* = 21) and 1.8% (*n* = 18), respectively (Online Supplement Table S1).

**Table 1:** Age, gender, smoking habits, symptoms and lung function values in 983 subjects according to different definitions of restrictive spirometry pattern (RSP) and true pulmonary restriction defined as TLC < LLN

	Restrictive spirometric pattern (RSP)			True pulmonary restriction	
	FEV_1_/FVC ≥ LLN and FVC < LLN (RSP_LLN_) *N* = 33 (3.4%)	FEV_1_/FVC ≥ 0.7 and FVC < 80% (RSP_0.7_) *N* = 31 (3.2%)	FEV_1_/FVC ≥ 0.7 and FVC < 80% predilation (RSP_predil_) *N* = 33 (3.4%)	TLC < LLN_5_ (Quanjer) *N* = 46 (4.7%)	TLC < LLN_5_ (Gothenburg) *N* = 53 (5.4%)
Males *n* = 500 (50.9%)	*n* = 19 (57.6%)	*n* = 17 (54.8%)	*n* = 18 (54.5%)	*n* = 39 (84.8%)	*n* = 28 (68.3%)
BMI (kg/m^2^)	29.3 (6.1)	29.8 (6.5)	29.2 (5.7)	28.8 (5.2)	28.6 (5.5)
Age (yrs)	57.0 (4.2)	57.9 (4.2)	56.8 (4.47)	57.8 (4.5)	56.9 (4.4)
Ever-smokers *n* = 570 (58.0%)	*n* = 21 (63.6%)	*n* = 17 (54.8%)	*n* = 20 (60.6%)	*n* = 25 (50.0%)	*n* = 21 (51.2%)
Never-smokers *n* = 417 (42.4%)	*n* = 12 (36.4%)	*n* = 14 (45.2%)	*n* = 13 (39.4%)	*n* = 25 (50.0%)	*n* = 20 (48.8%)
FEV_1_ (% pred)	76.1 (8.7)	75.7 (8.8)	78.8 (8.2)	82.9 (17.1)	84.4 (18.6)
FVC (% pred)	74.0 (6.6)	73.3 (6.3)	77.6 (7.3)	80.5 (17.7)	82.8 (19.2)
TLC_QUANJER_ (% pred)	90.3 (7.7)	90.7 (8.2)	89.6 (7.4)	74.6 (8.5)	73.0 (8.6)
TLC_GOTHENBURG_ (% pred)	84.9 (7.0)	85.9 (7.8)	84.1 (6.5)	77.5 (5.6)	77.8 (5.3)
RV (% pred)	99.2 (29.8)	99.9 (30.9)	100.4 (29.4)	84.5 (26.0)	82.6 (27.4)
Asthma *n* = 93 (9.5%)	*N* = 3 (9.1%)	*n* = 2 (6.5%)	*n* = 3 (9.1%)	*n* = 4 (8.0%)	*n* = 4 (9.8%)
MRC ≥ 2 *n* = 51(5.2%)	*N* = 5 (15.2%)	*n* = 5 (16.1%)	*n* = 5 (15.2%)	*n* = 4 (8.0%)	*n* = 5 (12.2%)
Diabetes *N* = 165 (16.7%)	*N* = 8 (24.2%)	*N* = 7 (22.6%)	*N* = 7 (21.2%)	*N* = 11 (23.9%)	*N* = 11 (20.8%)
Myocardial infarction *N* = 27 (2.8%)	*N* = 1 (3.1%)	*N* = 3 (10%)	*N* = 1 (3.0%)	*N* = 3 (6.7%)	*N* = 3 (5.7%)

Definition of abbreviations: *BMI* Body mass index, *FEV*_*1*_ Forced expiratory volume in one second, *FVC* Forced vital capacity, *LLN* Lower limit of normal, *TLC* Total lung capacity, *RV* Residual volume, *MRC* Medical Research Council

**Fig. 1:**
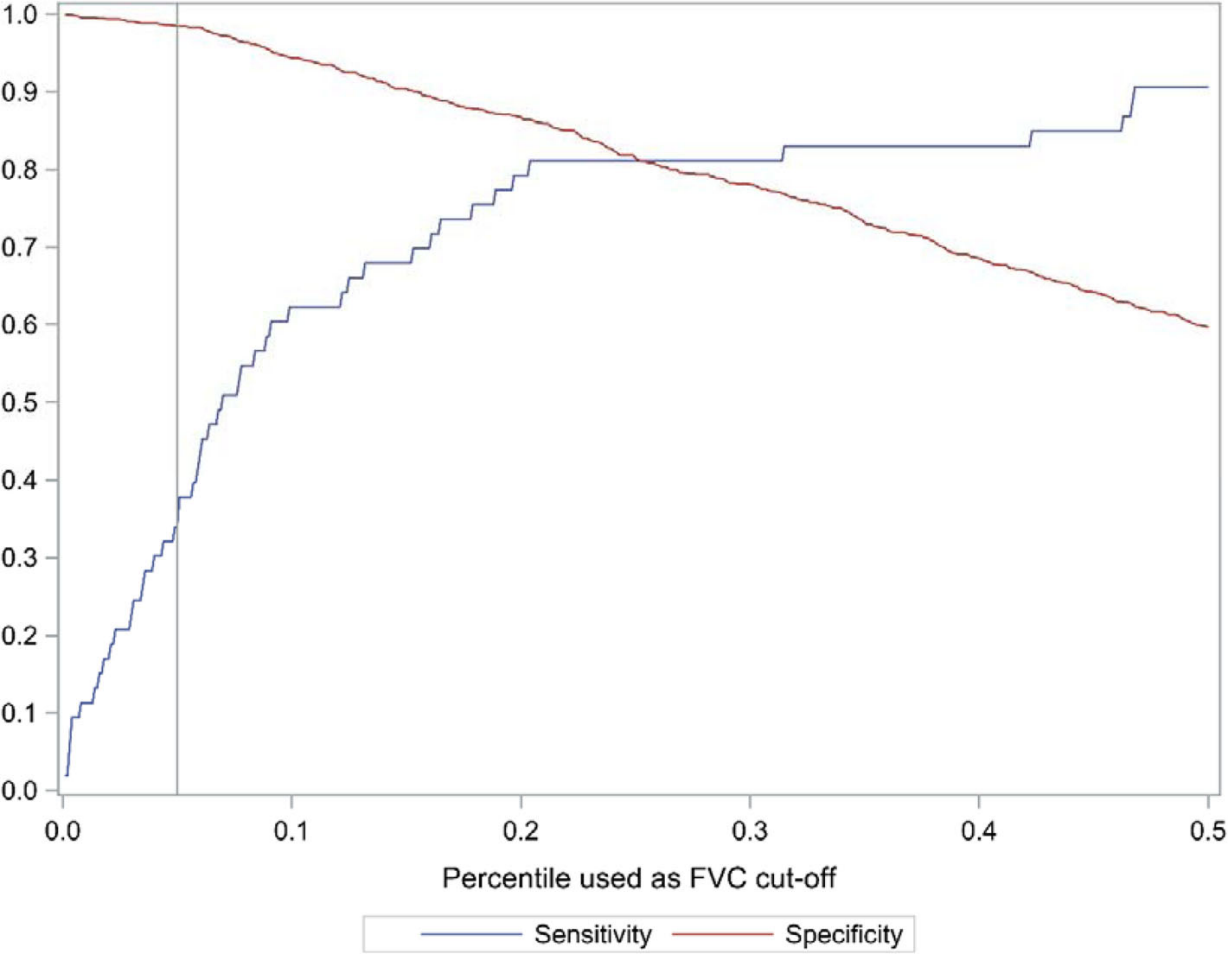
Sensitivity and specificity of restrictive spirometric pattern (RSP_LLN_) defined as FEV_1_/FVC ≥ LLN and FVC < LLN after bronchodilation using locally published Eqs. (17–18) in relation to true pulmonary restriction defined as TLC < LLN applying increasing percentiles (cut-off values) of FVC

The specificity, sensitivity, NPV and PPV, positive and negative likelihood ratio of RSP_0.7_, RSP_LLN_ and RSP_PREDIL_ in relation to true pulmonary restriction, defined either according to Quanjer et al. or based the Gothenburg equation, are shown in Table 2. When applying the Quanjer equation, the sensitivity of identifying TLC < LLN_QUANJER_, was about 0.30 for all definitions of RSP. The highest sensitivity, 0.33, was in relation to RSP_LLN_. The specificity was 0.98 for all definitions of RSP. All the RSP definitions has a high positive likelihood ratio, 17 to 24. With a pretest probability (prevalence) of true pulmonary restriction of 5.4%, a LR+ around 21 indicates a 50% post-test probability of true pulmonary restriction if presence of RSP. The negative likelihood ratios ranged from 0.67 to 0.71. A negative likelihood ratio around 0.70 means that there is a 4% post-test probability of true pulmonary restriction if there is no RSP. When applying the Gothenburg TLC equations to calculate predicted values the results were quite similar as compared to the Quanjer equation (Table 2). The sensitivity of identifying TLC < LLN_GOTHENBURG_ ranged from 0.32 to 0.36. A plot of increasing percentiles of FVC in the definition of RSP_LLN_ is shown in Fig. 1. Increasing the FVC from the 10th percentile to the 20th percentile increased the sensitivity from 0.55 to 0.80, with moderately decreased specificity. The max sensitivity and specificity was around 25th percentile of FVC.

**Table 2:** Validity of restrictive spirometric pattern (RSP) in relation to true pulmonary restriction

Restrictive spirometric pattern (RSP)	True pulmonary restriction			
	TLC < LLN_5_ (Quanjer) (*n* = 46)		TLC < LLN_5_ (Gothenburg) (*n* = 53)	
	Value	95% CI	Value	95% CI
RSP_LLN_ (*n* = 33)				
Sensitivity	0.33	0.20–0.48	0.34	0.20–0.46
Specificity	0.98	0.97–0.99	0.98	0.97–0.99
PPV	0.45	0.28–0.64	0.55	0.36–0.72
NPV	0.97	0.97–0.95	0.96	0.95–0.97
LR+	17.0	9.2–31.5	21.1	11.3–39.4
LR-	0.69	0.56–0.84	0.67	0.55–0.81
RSP_0.7_ (*n* = 31)				
Sensitivity	0.30	0.18–0.46	0.32	0.28–0.60
Specificity	0.98	0.98–0.97	0.98	0.97–0.99
PPV	0.45	0.27–0.64	0.55	0.36–0.73
NPV	0.97	0.95–0.98	0.96	0.95–0.97
LR+	16.8	8.8–31.9	21.3	11.1–40.9
LR-	0.71	0.59–0.86	0.69	0.57–0.83
RSP_PREDIL_ (*n* = 33)				
Sensitivity	0.30	0.18–0.46	0.36	0.23–0.50
Specificity	0.98	0.97–0.99	0.98	0.97–0.99
PPV	0.42	0.25–0.61	0.58	0.39–0.75
NPV	0.97	0.95–0.98	0.96	0.95–0.98
LR+	15.0	8.1–28.0	23.8	12.7–44.9
LR-	0.71	0.59–0.86	0.65	0.53–0.80

Definition of abbreviations: *LLN* Lower limit of normal; total lung capacity, *PPV* Positive predictive value, *NPV* Negative predictive value, *LR+* Positive likelihood ratio, *LR-* Negative likelihood ratio

When applying SVC, the specificity, sensitivity, NPV and PPV, positive and negative likelihood ratio of RSP_LLNSVC_ and RSP_0.7SVC_ in relation to true pulmonary restriction, defined either according to Quanjer et al. or based the Gothenburg equation, are shown in Table 3. Using SVC instead of FVC resulted in lower sensitivity and higher specificity, with no obvious differences between whether true pulmonary restriction was defined based on the Quanjer or the Gothenburg equation.

**Table 3:** Validity of restrictive spirometric pattern using slow vital capacity (RSP_LLNSVC_ and RSP_0.7SVC_) in relation to true pulmonary restriction

Restrictive spirometric pattern (RSP)	True pulmonary restriction			
	TLC < LLN_5_ (Quanjer) (*n* = 46)		TLC < LLN_5_ (Gothenburg) (*n* = 53)	
	Value	95% CI	Value	95% CI
RSP_LLNSVC_ (*n* = 33)				
Sensitivity	0.24	0.13–0.39	0.26	0.15–0.40
Specificity	0.99	0.98–0.99	0.99	0.98–1.00
PPV	0.52	0.30–0.74	0.67	0.43–0.85
NPV	0.96	0.95–0.97	0.96	0.94–0.97
LR+	22.4	9.2–31.5	35.1	14.8–83.3
LR-	0.77	0.56–0.84	0.74	0.63–0.87
RSP_0.7DSVC_ (*n* = 31)				
Sensitivity	0.22	0.11–0.36	0.21	0.11–0.34
Specificity	0.99	0.98–1.00	0.99	0.98–1.00
PPV	0.56	0.31–0.78	0.61	0.36–0.83
NPV	0.96	0.95–0.97	0.96	0.94–0.97
LR+	25.4	10.6–61.5	27.6	11.1–68.3
LR-	0.79	0.68–0.92	0.80	0.70–0.92

Definition of abbreviations: *LLN* Lower limit of normal; total lung capacity; *PPV* Positive predictive value, *NPV* Negative predictive value, *LR+* Positive likelihood ratio, *LR-* Negative likelihood ratio, *SVC* Slow vital capacity

Body mass index was positively associated with discordance between RSP and true pulmonary restriction (OR 1.10, 95% CI 1.03–1.17) and residual volume was negatively associated (OR 0.96, 95% CI 0.94–0.97) (Table 4).

**Table 4:** Logistic regression model of factors associated with disconcordance regarding true pulmonary restriction and restrictive spirometric pattern (RSP). All included co-variates are presented in the table

	Restrictive spirometric pattern (RSP)	
	RSP_LLN_	RSP_LLNSVC_
Co-variate	Odds ratio (95% CI)	Odds ratio (95% CI)
Age (yrs)	1.02 (0.95–1.09)	1.04 (0.97–1.12)
Body mass index (kg/m^2^)	1.10 (1.03–1.17)	1.10 (1.02–1.18)
Women vs. men	0.58 (0.31–1.06)	0.43 (0.22–0.84)
Eversmoking (Yes/No)	0.85 (0.47–1.55)	0.62 (0.33–1.17)
Residual volume (proc pred)	0.96 (0.94–0.97)	0.94 (0.93–0.96)

Definition of abbreviations: *LLN* Lower limit of normal, *SVC* Slow vital capacity, *CI* Confidence interval

## Discussion

The main result of the present study is that the sensitivity of RSP was fairly low in relation to true pulmonary restriction, whereas the specificity and NPV were high. The validity, sensitivity, and specificity were similar, regardless of which definition of RSP that was used. Finally, there was no difference between the pre- and post-bronchodilation results. Hence, we confirm previous results from referred-based populations, that RSP is relevant for ruling out true pulmonary restriction, and RSP has low validity in identifying true pulmonary restriction.

This is, to the best of our knowledge, the first study of a general population sample, although in a narrow age interval, sample validating RSP in relation to true pulmonary restriction, based on measurements of TLC by body plethysmography. Our results confirm results from previous clinical studies indicating a high NPV for RSP used as a proxy. This means that spirometry with no sign of RSP makes it highly unlikely that the person has true pulmonary restriction. When the prevalence of the true condition is low (< 5%), as in this case, the NPV rather accurately reflects that a negative test indicates no abnormality [23]. This conclusion is also supported by the low LR-. A high NPV (and low LR-) is especially valid in clinical situations where we have to consider whether to investigate a patient with lung volumes. In a clinical population, compared to a population study, the prevalence of true pulmonary restriction is probably higher. Hence, in the clinical situations the RSP may be more valid as a proxy for true pulmonary restriction. Still, the present results confirm earlier results that the proxy RSP could be used for ruling out true pulmonary restriction.

Even if the specificity was 0.98, this level of specificity of a test will generate a substantial number of false positives when applied in a general population study when the true condition, true pulmonary restriction, has a low prevalence. This will lead to falsely decreased risk estimates because of misclassification of the disease [24]. The conclusion from the present study is that RSP is a proxy for true pulmonary restriction with low validity. We made additional analysis using SVC instead of FVC. Not surprisingly, the prevalence of RSP decreased, the sensitivity decreased and the specificity went up to 0.99, further supporting previous conclusions.

We found that high BMI and low residual volume were associated with this disconcordance. There was also an association with disconcordance and male gender, although with borderline significance. The results were more obvious when using RSP_LLNSVC_ instead of RSP_LLN_. In clinical practice that means that overweight men may be at risk for disconcordant results. The material was too small to differentiate whether the disconcordance was dependent on true pulmonary restriction or on RSP.

The RSP phenotype has been linked to diabetes, metabolic syndrome and increased mortality [2]. The prevalence of RSP has mostly been below 10%, but in low and middle income countries prevalence figures between 25 and 69% have been reported. Some studies also report that RSP is more prevalent in older strata of the population [14, 25]. In a random population from Northern Sweden, the prevalence of RSP (after bronchodilation) was 5.4% in the age group 40 to 60 years [14]. Our study indicated a slightly lower prevalence, around 3%.

We used a published reference equation to estimate the predicted values of TLC [19]. In accordance with recommendations from the ATS/ERS Task Force, we also developed a local reference equation based on 354 healthy never-smokers [26]. These equations were developed using only height as a covariate, stratified for gender, applying the model described by Quanjer et al. [19]. The results were fairly similar, although the sensitivity was somewhat higher using the Gothenburg equations. The local equation did not add much to the results other than indicating that the published equation was suitable for our population. However, it has to be added that the present equations are not satisfactory as they are based on small populations. An equation for TLC and RV based on larger populations are highly warranted.

The main weakness of the present study is the small study sample. We have outlined 95% confidence interval around our estimates to be able to judge the reliability of our results. Another limitation is the narrow age interval 50–64 years, making the conclusions valid for this age group only. Selection bias may be a problem, as the participation rate was around 50%. In the current population chronic obstructive pulmonary disease and cardiovascular disease seem to have increased the participation, but we believe this has only marginally affected the validity of the estimates [27].

## Conclusion

RSP has low validity for identifying true pulmonary restriction. We do not recommend using RSP in general population studies as a proxy for true pulmonary restriction. Our results support previous observations that RSP is useful for ruling out true pulmonary restriction.

## Supplementary information

**Additional file 1: Table S1.** Age, gender, smoking habits, symptoms and lung function values in 983 subjects according to different definitions of restrictive spirometry pattern (RSP) based on slow vital capacity.

## Data Availability

The data is available upon request to the corresponding author. All analyses requires a permission from a Swedish ethical committee. A detailed description of the database can be found at www.scapis.org.

## Abbreviations

CI: Confidence interval
FEV_1_: Forced expiratory volume in one second
FVC: Forced vital capacity
LLN: Lower limit of normal
LR: Likelihood ratio
OR: Odds ratio
PREDIL: Prebronchodilation
RSD: Residual standard deviation
RSP: Restrictive spirometric pattern
RV: Residual volume
SCAPIS: Swedish CArdioPulmonary bioImage Study
SVC: Slow vital capacity
TLC: Total lung capacity
