# Supplementary material for: Restrictive spirometric pattern and true pulmonary restriction in a general population sample aged 50 - 64 years
Source: BMC Pulm Med. 2020 Feb 27;20:55. doi: 10.1186/s12890-020-1096-z (PMC7045734; doi:10.1186/s12890-020-1096-z)
Supplement: Supplementary file 1 — Additional file 1: Table S1. Age, gender, smoking habits, symptoms and lung function values in 983 subjects according to different definitions of restrictive spirometry pattern (RSP) based on slow vital capacity. [file 12890_2020_1096_MOESM1_ESM.docx]

**Online supplement.**

**Restrictive spirometric pattern and true pulmonary restriction in a general population sample aged 50 - 64 years**

Kjell Torén, Linus Schiöler, Jonas Brisman, Andrei Malinovschi, Anna-Carin Olin, Göran Bergström, Björn Bake

**Table S1. Age, gender, smoking habits, symptoms and lung function values in 983 subjects according to different definitions of restrictive spirometry pattern (RSP) based on slow vital capacity.**

|  | **Restrictive spirometric pattern (RSP_SVC_)** | |
| --- | --- | --- |
|  | FEV_1_/SVC≥LLN and SVC<LLN  (RSP_LLNSVC_)  N=21 (2.1%) | FEV_1_/SVC≥0.7 and SVC<80%  (RSP_0.7SVC_)  N=18 (1.8%) |
| Males  n=500 (50.9%) | n=13  (61.9%) | n=12  (66.7%) |
| BMI (kg/m^2^) | 28.6 (6.3) | 29.9 (7.1) |
| Age (yrs) | 57.5 (4.3) | 58.9 (4.6) |
| Ever-smokers  n=570 (58.0%) | n=12  (57.1%) | n=9  (50.0%) |
| FEV_1_  (% pred) | 73.6 (9.2) | 72.0 (8.8) |
| FVC  (% pred) | 71.8 (7.1) | 70.4 (6.5) |
| TLC_QUANJER_  (% pred) | 81.0 (8.3) | 79.1 (8.3) |
| TLC_GOTHENBURG_  (% pred) | 79.1 (6.4) | 78.2 (7.2) |
| RV  (% pred) | 98.9 (31.5) | 103.4 (32.5) |
| Asthma  n=93 (9.5%) | N=3  (14.3%) | n=1  (5.6%) |
| MRC≥2  n=51(5.2%) | N=3  (14.3%) | n=4  (22.2%) |
| Diabetes  N=165 (16.7%) | N=3  (14.3%) | N=4  (22.2%) |
| Myocardial infarction  N=27 (2.8%) | N=0 | N=3  (17.6%) |

Definition of abbreviations: BMI=body mass index; FEV_1_=forced expiratory volume in one second; FVC=forced vital capacity; LLN=lower limit of normal; SVC=slow vital capacity; TLC=total lung capacity; RV=residual volume; MRC=Medical Research Council;
